# The Paradox in AI Influencer Engagement: A Dual Path to Psychological Need Satisfaction and Frustration

**DOI:** 10.3390/bs16040610

**Published:** 2026-04-20

**Authors:** Ha Eun Park

**Affiliations:** Department of Big Data Analytics, Kyung Hee University, Seoul 02447, Republic of Korea; gracepark129@khu.ac.kr

**Keywords:** artificial intelligence, AI influencer, AI-mediated engagement, self-determination theory, psychological need satisfaction, need frustration, human-AI interaction

## Abstract

As AI-generated influencers increasingly dominate social media landscapes, their psychological impact on human users necessitates rigorous empirical investigation. Grounded in Self-Determination Theory, this study examines how AI influencers influence the satisfaction and frustration of users’ basic psychological needs—autonomy, competence, and relatedness. Utilizing a netnographic approach, the research identifies three pivotal psychological mechanisms. The findings reveal a fundamental paradox characterized by a dual-path process; while AI influencers can meaningfully fulfill psychological needs through consistent presence and customizable narratives, they simultaneously risk undermining these needs when perceived as instruments of algorithmic surveillance, commercial orchestration, or emotional inauthenticity. This duality underscores the complexity of AI-mediated engagement, where the same technological affordances can lead to either psychological flourishing or digital alienation. These insights emphasize the urgency for responsible AI design that prioritizes user well-being over mere commercial conversion, offering critical implications for developers, marketers, and policymakers in the evolving era of AI-driven social interaction.

## 1. Introduction

The rapid diffusion of artificial intelligence (AI) into everyday digital environments is fundamentally reshaping how individuals experience social interaction, identity construction, and psychological well-being. One prominent manifestation of this shift is the emergence of AI influencers, AI-generated virtual personas that simulate human appearance, emotions, and social behaviors on social media platforms (Association of National Advertisers, 2018). While originally introduced as marketing tools within influencer ecosystems, AI influencers increasingly function as social actors with whom users interact, emotionally engage, and form ongoing relationships. AI influencers are commonly defined as AI-generated virtual humanoid entities that emulate human appearance and behavior in social media contexts (Feng et al., 2024). Unlike traditional social media influencers, these entities are algorithmically designed, visually immutable, and strategically curated to deliver consistent narratives, emotions, and interactions at scale. High-profile examples such as Lil Miquela, a virtual influencer with millions of followers and collaborations with global brands (Alexander, 2019; da Silva Oliveira & Chimenti, 2021; Pantaleon, 2019), illustrate how AI-generated personas can become embedded in users’ everyday digital routines, occupying spaces traditionally reserved for human social figures. As a result, interactions with AI influencers increasingly extend beyond commercial exposure to include identity projection, emotional engagement, and perceived social connection.

Existing research suggests that AI influencers often generate high levels of engagement and positive user responses (Baklanov, 2021; Mileva, 2024). Their perceived advantages, such as consistency, availability, and controllability, make them attractive alternatives to human influencers. However, a focus on engagement metrics and outcomes obscures a more fundamental question: how do interactions with AI-generated social agents shape users’ psychological experiences and motivational states? The critical issue is not whether AI influencers are effective communicators, but how they alter the satisfaction and frustration of basic psychological needs that underpin psychological well-being. Although AI influencers are designed to resemble humans, they differ from human influencers in important psychological ways. AI influencers are digitally constructed entities whose emotions, expertise, and social interactions are pre-scripted, optimized, and algorithmically orchestrated (Choudhry et al., 2022; Moustakas et al., 2020; Thomas & Fowler, 2021). These characteristics create a unique interaction context in which users may simultaneously experience both the fulfillment and frustration of autonomy, competence, and relatedness due to inherent asymmetries in AI-mediated interactions. Consequently, user engagement with AI influencers may involve ambivalent psychological outcomes, rather than uniformly positive experiences.

Despite growing scholarly attention to AI influencers, existing studies have largely examined users’ trust, acceptance, and attitudinal responses (e.g., Alboqami, 2023; De Cicco et al., 2024; Feng et al., 2024; Sands et al., 2022b), as well as brand-related implications (Thomas & Fowler, 2021). While these contributions are valuable, they provide limited insight into how AI influencers affect users’ intrinsic motivation and psychological well-being. In particular, little is known about how interactions with AI influencers simultaneously support and undermine fundamental psychological needs, such as autonomy, competence, and relatedness. Recent calls have emphasized the need to move beyond surface-level evaluations of AI influencers and toward deeper examinations of their psychological well-being (Sorosrungruang et al., 2024).

Drawing on self-determination theory (SDT) and a netnographic analysis of AI influencer interactions on Instagram, this study examines how AI influencers shape users’ psychological experiences by simultaneously satisfying and frustrating their basic psychological needs. Rather than conceptualizing AI influencers merely as communication or marketing tools, this research approaches them as AI-mediated social agents that participate in users’ motivational ecosystems. Specifically, the study addresses the following research question: How do AI influencers simultaneously satisfy and frustrate users’ needs for autonomy, competence, and relatedness, and what are the psychological well-being implications of these paradoxical processes? To answer this question, the study develops an empirically grounded framework that explicates the psychological mechanisms through which AI influencers influence users’ experiences of autonomy, competence, and relatedness. In doing so, it extends SDT to the context of AI-mediated social interaction by demonstrating how the same design and communication features of AI influencers can generate both need satisfaction and need frustration. Inspired by prior research from Marriott and Pitardi (2024) on AI friendship that highlights the paradoxical coexistence of perceived emotional support and diminished well-being, this study identifies both the empowering and potentially undermining implications of AI influencer engagement. Finally, the findings offer practical implications for AI influencer designers, brands, and social media strategists by outlining design and engagement approaches that balance user engagement with psychological well-being.

## 2. Conceptual Background

### 2.1. Social Media Influencers

Research on social media influencers (SMIs) has extensively examined how individuals engage with influential figures in digitally mediated social environments. Rather than functioning merely as sources of information or persuasion, SMIs have been shown to operate as social actors who shape users’ psychological experiences through ongoing interaction, identification, and emotional engagement (Lou & Yuan, 2019). Prior research has highlighted several key mechanisms underlying these interactions, including source credibility, perceived alignment between the influencer and endorsed content, and parasocial relationships (Hudders et al., 2021). Importantly, these mechanisms provide a foundational understanding of how influencer–user interactions can support users’ motivation, sense of self, and social connectedness.

Source credibility has been a central construct in SMI research and refers to the perceived qualities of a communicator that influence message reception (Ohanian, 1990). Within influencer contexts, credibility is commonly understood as comprising perceived attractiveness, expertise, and trustworthiness. These attributes shape users’ willingness to attend to, internalize, and emotionally engage with influencer content (Reinikainen et al., 2020). From a psychological perspective, perceived credibility does not merely facilitate persuasion but also contributes to users’ sense of confidence and reassurance when navigating social and informational environments. What matters is therefore not the influencer’s objective expertise or authenticity, but users’ subjective perceptions, which can influence their feelings of confidence and certainty in decision-making processes.

Another stream of research has examined perceived alignment between influencers and the content they present, often conceptualized as product–endorser fit or match-up (De Cicco et al., 2021; H. J. Park & Lin, 2020; Schouten et al., 2020). While this literature has traditionally emphasized advertising effectiveness, it also reveals important psychological dynamics. When influencers are perceived as congruent with the values, lifestyles, or domains they represent, users are more likely to view interactions as coherent and meaningful. Such perceived coherence can reduce cognitive dissonance and support users’ sense of understanding and mastery within mediated environments. Conversely, perceived misalignment may disrupt users’ trust and undermine their psychological comfort (Lee & Koo, 2015), highlighting how influencer design and presentation can shape users’ motivational experiences.

Parasocial interaction refers to the experience of a one-sided, yet subjectively meaningful, relationship with a media figure (Schmid & Klimmt, 2011). Repeated exposure and interaction can foster parasocial relationships characterized by feelings of familiarity, affection, and emotional attachment (Tukachinsky, 2010). Research has shown that such relationships can enhance users’ enjoyment of content, foster emotional bonds, and create a sense of social presence and belonging (Hartmann & Goldhoorn, 2011; Ko & Wu, 2017; Munnukka et al., 2019). Parasocial relationships illustrate how mediated figures can partially fulfill socialization, even in the absence of reciprocal interaction.

Collectively, prior research on SMIs demonstrates that influencer–user interactions are not purely instrumental but are intertwined with users’ psychological experiences. However, this literature has largely assumed human agency, lived experience, and authentic emotional capacity on the part of influencers. These assumptions become increasingly problematic in the context of AI-generated influencers, whose social cues, emotions, and interactions are algorithmically constructed rather than humanly enacted. As such, while SMI research offers an essential theoretical foundation for understanding influencer-based social interaction, it is insufficient for explaining the psychological dynamics that emerge when influencers are no longer human, a gap that the following section on AI influencers seeks to address.

### 2.2. AI Influencers

AI influencers differ fundamentally from human influencers in that they are artificially generated social entities whose appearance, emotions, narratives, and interactions are algorithmically designed rather than experientially lived. AI influencers differ conceptually from traditional digital agents and other forms of algorithmically mediated interactions through their unique combination of autonomous control and complex humanized narratives. While standard digital agents (such as basic chatbots) often serve functional, task-oriented purposes, an AI influencer is a human-like entity that is autonomously controlled by AI and visually presented as an interactive, real-time rendered being in a digital environment (Sands et al., 2022b). AI allows them to simulate real life and a personality and interactions that all feel natural. The details of AI influencers’ imaginary lives, very realistic in the long term, help to generate a lasting attachment and identification in their audiences (Allal-Chérif et al., 2024). Despite their non-human nature, AI influencers are often perceived as highly human-like due to advanced visual rendering, expressive facial features, stylized bodies, and emotionally framed communication presented through social media posts and interactions (Choudhry et al., 2022). As a result, users may engage with AI influencers in ways that closely resemble interactions with human social actors, blurring the boundary between artificiality and perceived social presence.

A defining feature of AI influencers is anthropomorphism, or the attribution of human characteristics, such as emotions, intentions, personality, and social agency, to non-human entities (Baek et al., 2022). Prior research conceptualizes AI influencers as humanized or anthropomorphic digital agents whose design is intended to facilitate natural, intuitive, and emotionally engaging interactions with users (da Silva Oliveira & Chimenti, 2021; Hoffman et al., 2021). Through human-like facial expressions, conversational language, and emotionally resonant narratives, AI influencers are designed to evoke familiarity, trust, and social connection. Anthropomorphism serves not merely as a design feature but as a mechanism that invites users to interpret AI influencers as intentional social beings, capable of emotional understanding and relational engagement.

Emerging research on AI influencers has primarily examined users’ attitudinal and evaluative responses, including trust formation (Alboqami, 2023), consumer reactions and acceptance (Arsenyan & Mirowska, 2021; Sands et al., 2022a), perceived influencer attributes (Block & Lovegrove, 2021; Feng et al., 2024), and comparative perceptions of AI versus human influencers (De Cicco et al., 2024; J. Park et al., 2024). Collectively, these studies have provided valuable insights into when and why users respond favorably to AI influencers and how such responses may benefit brands (Thomas & Fowler, 2021; see Table 1 for more details). However, this body of work remains largely outcome-oriented, focusing on surface-level evaluations rather than the underlying psychological processes through which AI influencers shape user experience. Critically, existing research has paid limited attention to the psychological implications of interacting with AI influencers as social agents. While anthropomorphism and human likeness are often framed as drivers of engagement and acceptance, far less is known about how these features shape users’ intrinsic motivation and psychological needs. Interactions with AI influencers may support users’ experiences of autonomy, competence, and relatedness by enabling self-expression, perceived guidance, and emotional connection. At the same time, the algorithmic and non-reciprocal nature of AI influencers introduces structural asymmetries, such as the absence of genuine agency, emotional reciprocity, and lived experience, that may frustrate these same psychological needs. This duality suggests that AI influencer engagement is not uniformly beneficial but may involve paradoxical psychological dynamics.

While scholars have started to investigate AI influencers, existing studies are fragmented, and little is known about the impacts of AI influencers on social media users. While these studies discuss the factors that influence customers’ acceptance and trust in AI influencers, it is vital to explore how AI influencers impact users’ intrinsic motivation—their psychological needs—to understand deeper AI influencer–user engagement. This research investigates this phenomenon by employing self-determination theory as a theoretical lens in the subsequent section.

### 2.3. Self-Determination Theory and Paradox

To explore how AI influencers on social media influence users’ psychological needs, this research turns to self-determination theory (SDT; Deci & Ryan, 1985), a macro-theory for understanding human motivation (Kosa & Uysal, 2022). SDT describes “a set of psychological needs whose satisfaction is an intrinsically motivating source of action, which provides energy for individuals to act on their environment and manage their behaviors in a self-determining fashion” (Deci & Ryan, 2000). SDT identifies two types of motivation, which are intrinsic and extrinsic. Intrinsic motivation arises when the values underlying a behavior align with an individual’s inherent satisfaction and enjoyment (Groening & Binnewies, 2021), and extrinsic motivation occurs when an individual engages in an activity driven by external rewards or consequences (R. Chen et al., 2025). People are naturally inclined to grow, develop, and attain psychological needs to unify a sense of self and integrate them into society; however, this can be achieved when they satisfy three fundamental psychological needs: competence, autonomy, and relatedness (Deci & Ryan, 1985; Ryan & Deci, 2017).

In SDT, autonomy refers to an individual’s inherent need to feel self-directed, regulate their own actions, and maintain a sense of control. Autonomy acts as a crucial intrinsic motivation, where individuals engage in activities because they perceive them as inherently satisfying or interesting (Laato et al., 2022). Competence reflects the need to feel capable and effective in interactions with the environment, associating with the experience of mastery (Kosa & Uysal, 2022). Lastly, relatedness encompasses the need to feel connected, engaged with others, and experience a sense of belonging, developing meaningful connections with others (Ryan & Deci, 2017). When these psychological needs are fulfilled, individuals are inclined to experience benign well-being and motivation (Wang et al., 2025), and therefore, needs satisfaction is pivotal for enhancing intrinsic motivation and accomplishing optimal well-being (Barberis et al., 2022; Ryan & Deci, 2017).

While recent studies employing SDT in the context of social media have yielded valuable insights, they have predominantly focused on enterprise social media (e.g., Sun et al., 2025) and the influence of psychological needs on the use of social media applications (e.g., Karahanna et al., 2018). In terms of enterprise social media, Wei et al. (2022) examined how three basic psychological needs affect employee social media use. Sun et al. (2025) investigated how enterprise social media affordances affect employee agility through the three basic psychological needs satisfaction. Tsai et al. (2021) investigated how intrinsic motivations affect exercisers’ and spectators’ perceptions using real-time interactive mobile running applications. Regarding the impact of needs on social media use, Karahanna et al. (2018) identified that psychological needs motivate the use of social media applications. While several studies have contributed to understanding users’ psychological needs in the context of social media, they primarily focus on enterprise social media and the use of social media. Given the growing popularity of AI influencers, there is a clear need for more research on how AI influencers on social media influence users’ psychological needs, such as autonomy, competence, and relatedness. This study explores the psychological mechanisms behind how AI influencers shape the psychological needs of social media users. Understanding these dynamics is crucial for optimizing social media engagement to enhance individual well-being.

In the realm of motivational frameworks, paradox theory intersects meaningfully with SDT by highlighting the dynamic frictions that can influence intrinsic and extrinsic drivers of behavior. Paradox theory has gained prominence as a framework for dissecting the built-in conflicts stemming from elements that appear incompatible yet are fundamentally linked in evolving and intricate systems (Smith & Lewis, 2011). It underscores the simultaneous presence of clashing dynamics, where divergent pressures demand ongoing adaptation from entities involved. Paradox theory characterizes a paradox as “opposing yet connected components that coexist” (Smith & Lewis, 2011, p. 386) that endures and invites integrative “both/and” tactics (Smith, 2014). The essential traits of a paradox include oppositions, where the components clash and produce inherent strains, and linkage, where the conflicting parts rely on each other, with one gaining vitality from the other’s presence (Smith & Lewis, 2011). Smith (2014) posits that effective handling begins with accepting that conflicts will linger indefinitely without final closure, repositioning them as an integral feature of systemic existence to be perpetually overseen. Navigating paradoxes necessitates transcending binary logic toward holistic approaches and this involves perpetual balancing of rival needs, fostering more adaptable structures, and generating mutual enhancements among the clashing aspects.

## 3. Research Method

Netnography was considered ideal for this research due to its emphasis on human and AI influencer interactions in digital spaces (i.e., social media). Following Kozinets’s (2020) procedural framework, the study progressed through six stages: initiation (research preparation), investigation (data collection), interaction (data collection), immersion (data collection), integration (data analysis), and incarnation (data presentation). Given the researcher’s passive role as an observer on social media, the interaction phase merged with immersion. Instagram was selected as the research context as it is among the most profitable platforms for influencer marketing (Laestadius, 2016). Since the data were retrieved from publicly accessible Instagram profiles of AI influencers, entities with negligible perceived risks, the research was conducted without direct interaction with users to avoid disrupting the natural social ecosystem. Adhering to Kozinets’s (2020) guidelines, this study implemented an anonymization strategy as ethical safeguards. All usernames and pseudonyms were removed, and verbatim quotes were subjected to subtle modification with synonym replacement to prevent back-tracing via search engines, thereby protecting user privacy in the public domain.

### 3.1. Data Collection

Netnographic data collection involved immersion and investigation (Kozinets, 2020). Immersion entails the researcher fully engaging in the project’s conceptual space. Its purpose was to uncover nuanced cultural insights regarding the portrayal of virtual entities on social media and the reactions of both the audience and the media. The researcher’s use of Instagram since 2014, including following SMIs, engaging with their content, and curating posts, contributed to over ten years of immersion on the platform. This prolonged engagement is crucial as it allows the researcher to view the phenomenon from an insider’s perspective, enhancing their understanding of the research content.

The data collection and observation spanned 1.5 years from May 2024 to December 2025. The netnographic immersion commenced in May 2024, with a focus on exploring the history of AI influencers’ posts and the interactions between them and their followers and audiences. This extended immersion fostered a comprehensive understanding of the phenomenon and offered the researcher the opportunity to examine intriguing trends and patterns in AI influencers’ posts and comments. A set of keywords and phrases was compiled to track relevant online sources. The keywords used were, for example, “AI influencers”, “virtual influencers”, “artificial intelligence”, “digital humans”, and “virtual avatars”. Consequently, various online materials related to AI influencers, including news articles, journal publications, websites, and other social media platforms, were analyzed to enhance the researcher’s understanding of AI influencers on social media. By integrating multiple data sources, the researcher could gain a deeper understanding of the evolving phenomenon of AI influencers (Kozinets, 2020). The immersion on Instagram focused on exploring AI influencers’ Instagram accounts and posts, including short videos, images, and comments.

In the investigation phase, to answer the research question, the selection criteria were developed to identify appropriate AI influencers for this study (Kozinets & Seraj-Aksit, 2024). First, as the scope of the present study focuses on AI influencer–user engagement to explore its impact on users’ psychological needs, the researcher identified AI influencers involved in endorsements or promotions on social media platforms such as Instagram and TikTok. Second, the focus of the content was mainly on the AI influencer rather than the creator. This emphasis stemmed from the research objective, which aimed to examine how AI influencers impact users’ psychological needs. After applying these selection criteria, the most-followed AI influencers within the virtual influencer category were identified for further analysis. To ensure a comprehensive and representative scope, this study specifically analyzed high-profile AI influencers with diverse global and regional orientations: Lil Miquela (global/USA), Lu do Magalu (Brazil), Rozy (Korea), Noonoouri (Europe/Fashion), and Sophia the Robot (AI and Fashion). These entities were selected based on their high follower counts, active participation in brand endorsements, and consistent user interaction levels on Instagram. The interactions monitored spanned communication and asynchronous social exchanges. Specifically, the research examined user-generated comments in response to posts, product breakdowns, and participation in social movements (e.g., #BeYourOwnRobot or environmental activism) to capture the nuances of parasocial bonding and reciprocity failure.

To gain a deeper understanding of the data, the set of posts with the most likes and comments was identified (Reid & Duffy, 2018). The subset of posts was then selected to provide a rich blend of source and content elements. Only publicly visible posts and comments were collected. The researcher retrieved available posts from the AI influencers’ respective social media pages. While data formats varied, specifically short-form videos and a combination of images, texts, carousels, and Reels on Instagram, the retrieval process focused on capturing the content and associated follower interactions and comments for each influencer. To ensure a manageable yet representative sample size, the most-liked and most-commented posts from each influencer were selected, as high engagement levels correlate with the highest density of diverse user responses. By prioritizing posts with the highest social proof, the research captures the most prominent and visible instances.

To systematically capture the psychological nuances of user engagement, this study utilized the theoretical dimensions of the Basic Psychological Need Satisfaction and Frustration Scale (BPNSFS) (B. Chen et al., 2015) as the primary analytical framework for data collection and thematic coding. Unlike traditional SDT approaches that focus solely on need satisfaction, the BPNSFS allows for a multidimensional exploration of both the growth-promoting (satisfaction) and the distressing (frustration) pathways of user experience (Vansteenkiste et al., 2020). During the netnographic analysis, user comments were categorized based on linguistic markers that aligned with these two distinct poles. Comments reflecting volitional engagement and mastery were coded as need satisfaction, while those expressing feelings of being manipulated or outperformed by AI were coded as need frustration, providing the empirical basis for the hedonic paradox (B. Chen et al., 2015; Ryan & Deci, 2017).

### 3.2. Data Analysis

This study adopted the SDT as the sensitizing device (Klein & Myers, 1999), providing a theoretical lens to guide the interpretation of psychological needs, autonomy, competence, and relatedness in social media interactions. The first step in the analysis involved data organization and familiarization. All collected digital texts, including social media posts, comments, and content, were compiled and categorized based on the research question. The dataset was reviewed multiple times to gain an initial understanding of emergent patterns and contextual nuances.

The coding process progressed through three stages, open, axial, and selective coding (Strauss & Corbin, 1998), and was managed using NVivo 15 software. The use of NVivo facilitated the systematic organization of netnographic data, allowing for the efficient categorization of themes and the identification of relationships between AI influencer content and follower responses. Open coding involved segmenting the data into discrete meaning units, with initial codes assigned to fragments of text representing specific ideas, behaviors, or sentiments. The study analyzed a corpus consisting of approximately 150 social media posts and 1000 associated follower comments from 5 prominent AI influencers. These influencers were selected from diverse areas of activity, including fashion (e.g., Lil Miquela), beauty (e.g., Lu do Magalu), and lifestyle (e.g., Rozy), ensuring that the findings reflect a broad cross-section of the AI influencer ecosystem. Codes (e.g., first-order concepts) were generated, ensuring that themes emerged organically from the data rather than being pre-imposed. For example, one of the first-order concepts included that AI influencers have human-like appearance, emotions, and expressions (retrieved from one of the users’ excerpts of “Aren’t you hot? Your skin will be burnt by the sun”). Axial coding involved exploring relationships among the initial codes to identify second-order themes (e.g., Immutable appearance). Linkages between codes were visualized through hierarchical mapping, enabling the identification of broader themes. Selective coding involved developing the central storyline of the study by integrating major themes into a coherent framework. Aggregate dimensions were identified that encapsulated the essence of digital discourse (e.g., Symbolic Play).

A challenge in analyzing digital discourse is the potential for performative behavior and social desirability bias. To ensure the validity of the psychological interpretations, this study employed prolonged netnographic immersion (spanning 1.5 years), allowing the researcher to distinguish between fleeting trends and consistent psychological patterns. Furthermore, the analysis accounted for the pact of fiction, the unique social contract where users knowingly engage with a virtual entity. By triangulating user comments with diverse data sources, including multiple AI influencers with diverse global and regional orientations, news articles, and blogs, the research moved beyond surface-level likes to analyze linguistic markers. Crucially, the analysis identified that expressions of the paradoxical experience, where users grapple with the tension between empowerment and algorithmic surveillance, often manifest in less performative, more visceral linguistic behaviors compared to the standardized praise found in promotional threads. These moments of ontological friction provide a more reliable window into the user’s genuine psychological state, as they deviate from the platform-driven performance of typical fan engagement. This approach ensures that the analyses reflect psychological responses to AI-mediated agency rather than mere platform-driven performance (Kozinets & Seraj-Aksit, 2024).

To enhance analytical clarity, the visual mapping technique was employed throughout the coding process. Concept maps and cluster diagrams were used to illustrate the relationships among themes, showcasing key narrative trajectories within the dataset. These visual tools facilitate the identification of thematic convergence and divergence. The final phase of analysis employed narrative strategies (Langley, 1999), enabling a dynamic representation of the data. A storyline was developed to illustrate how themes interconnect within the social media platform, capturing patterns of AI influencer–user engagement.

To enhance the rigor of the research, findings were compared across different social media platforms, news articles, blog websites, and data sources. The first coder conducted the initial round of open coding, identifying emerging themes and patterns in the dataset. To establish trustworthiness in data interpretation, a second coder was involved and cross-verified patterns to minimize individual bias and to validate the emerging themes. A second coder independently reviewed the data, cross-checking the initial codes against the original dataset. Any discrepancies or alternative interpretations were discussed to ensure coding consistency and mitigate individual biases. Both coders engaged in iterative discussions to refine and validate the coding framework, ensuring thematic coherence. Where disagreements arose, the coders revisited the data, leading to an agreed-upon coding structure (Malterud, 2001). Analytical consistency was maintained by revisiting coded data at multiple points in the study. The process of data analysis was juxtaposed with existing literature and continued until the point of theoretical saturation was reached.

## 4. Findings

**Figure 1:**
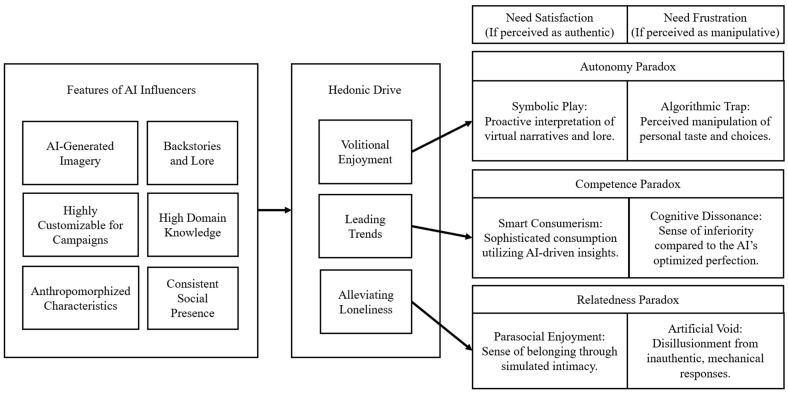
The Theoretical Framework of the Paradox in AI Influencer Engagement: A Dual-Path Model of Psychological Need Satisfaction and Frustration.

This study investigates how interaction with AI influencers on social media contributes to both the satisfaction and frustration of users’ basic psychological needs for autonomy, competence, and relatedness. Grounded in a netnographic analysis, the findings identify three psychological mechanisms that drive paradoxical implications. The empirically grounded theoretical framework, illustrated in Figure 1, demonstrates how each mechanism operates through a dual-path process. Each pathway begins with the unique features of AI influencers and is catalyzed by the user’s hedonic drive, ultimately resulting in either need satisfaction or need frustration depending on the user’s perception of authenticity versus algorithmic control (see Table 2 for more details).

The paradoxical reactions frequently emerge within the same users’ comments, in addition to separate groups of users expressing opposing views. These supports interpreting the patterns as a psychological paradox (e.g., emotional attachment to a constructed persona alongside cognitive dissonance about its artificial nature). Data analysis reveals that comments often blend expressions of need satisfaction, such as emotional attachment (“I believe you”), esthetic admiration (“the robot more pretty”), or investment in personal narratives, with simultaneous need frustration or cognitive dissonance regarding the influencer’s virtual status (“I know this is crazy,” “are you real?”). This intra-individual mixing reinforces the interpretation of a psychological paradox, where the follower maintains a meta-awareness of the influencer’s robotic nature while volitionally pursuing parasocial fulfillment. Such “both/and” logic (Smith, 2014) suggests that users formed bonds with virtual entities while retaining a recognition of their artificiality (Allal-Chérif et al., 2024; Block & Lovegrove, 2021).

### 4.1. The Autonomy Paradox: From Symbolic Play to the Algorithmic Trap

The data analysis reveals that the impact of AI influencers on user autonomy is initiated by the core features of AI influencers, such as their AI-generated imagery, backstories, and lore. These attributes serve as the foundation for the user’s hedonic drive, specifically the desire for volitional enjoyment and creative exploration within a virtual space. This drive activates the central mechanism of symbolic play, where the AI’s anthropomorphized characteristics and predefined backstories and lore ironically function as a structured yet open symbolic canvas, where users treat the AI’s esthetic as a playground for self-expression. Because AI is a non-human entity with a consistent aesthetic, users feel a sense of agency in proactively projecting their personal values and imaginative interpretations onto the influencer’s identity.

According to SDT and the BPNSFS framework (B. Chen et al., 2015), for autonomy, the data showed users exercising volitional engagement by choosing how and when to interact, often framing their participation as self-directed exploration rather than obligation. Examples include users stating “I come here when I need inspiration without any pressure,” which highlights a sense of choice. Virtual influencers’ controlled yet flexible personas appeared to support autonomous motivation by offering customizable engagement (e.g., users selectively responding to certain content types) without the interpersonal demands sometimes present in human influencer interactions. Conversely, occasional comments expressing discomfort with overly scripted content (“It feels too perfect to relate to”) highlighted potential autonomy-thwarting elements when authenticity perceptions were low. This process facilitates autonomy satisfaction; users perceive their interaction not as a passive reception of content, but as a self-endorsed act of meaning-making. For example, users often express that AI’s nature allows them to co-author the narrative. The mechanism of symbolic play represents a unique psychological process where users transform the inherent limitations of AI influencers into opportunities for autonomous engagement. Within this framework, the AI-generated appearance and predefined backstories of AI entities do not function as rigid constraints; rather, they serve as a stable semiotic anchor that invites user participation. Because an AI influencer lacks a true biological consciousness, it exists as a symbolic vessel. This ontological void allows users to engage in a form of imaginative projection, where they proactively fill the gaps of the virtual persona with their own desires, values, and creative narratives.

Drawing on the hedonic drive for volitional enjoyment, symbolic play occurs when users treat the AI’s consistent esthetic as a playground for self-expression. In this process, the user is no longer a passive recipient of a marketing message but becomes an active co-creator of the influencer’s social meaning. This proactive appropriation of the AI’s identity constitutes a high level of psychological agency, as the user dictates the terms of the interaction and the truth of the character. Consequently, this leads to autonomy satisfaction, as the experience fulfills the basic psychological need to feel like the origin of one’s own actions (Ryan & Deci, 2017). By playing with the symbolic boundaries of AI, users experience a sense of symbolic play, a volitional state where they feel empowered to navigate and redefine the virtual narrative according to their own internal values. Lil Miquela’s Instagram often features content and shares posts where she experiments with fashion, music, and activism that encourage self-expression and individuality. For example, in various posts, she showcases diverse fashion styles and artistic endeavors, inspiring followers to embrace their uniqueness. In her post with the caption “Rewriting my story every day #BeYourOwnRobot,” she wears an eclectic outfit that breaks conventional beauty norms. Her followers have responded positively to such content, with comments like: “You inspire me to wear what I want and not care what people think [user ID: low***],” “Finally someone (even if AI) showing real creativity in style! [ver***],” “Your style is so unique! You’ve inspired me to try new things [sah***],” and “Love how you express yourself. Makes me feel more confident to be me [wyn***].”

However, this satisfaction path is inherently countered by a parallel path of frustration when the same AI features are perceived through the lens of underlying control. The customizability that allows for creative campaigns can be sensed as a form of algorithmic trap, where every interaction is precisely engineered to maximize commercial conversion. When users become aware that their volitional engagement is being monitored and steered by data-driven optimizations, the hedonic drive is stifled, and the mechanism shifts toward the algorithmic trap. In this state, the sense of being a self-directed agent is replaced by the feeling of being a targeted data point, leading to autonomy frustration. While users are generally aware of the influencers’ AI nature, they often engage in a voluntary suspension of disbelief, entering a social frame where they interact with the digital persona as a relatable entity. The transition from satisfaction to frustration often occurs at the breaking of the frame, which occurs when the influencer’s output becomes incongruent with its established persona, specifically when commercial motives or uncanny robotic behavior overwhelm the social narrative. This disruption is often triggered by overly generic promotional language, a lack of contextual emotional depth, or highly stylized corporate captions that fail to mirror the authentic vernacular of the platform. In promotional posts or heavily stylized content, such as brand collaborations, followers express discomfort or skepticism. For example, a caption like “Just vibing in the metaverse with my favorite humans #ad” can trigger mixed reactions by highlighting the artificiality of the interaction. In discussions from Lil Miquela’s followers, comments include: “This feels like a corporation trying to be quirky… I miss real people [lor***],” and “It’s weird that you post as if you have real emotions. Who’s really behind this? [the***].”

Thus, the AI influencer design presents a fundamental paradox: it offers a platform for symbolic liberation while simultaneously acting as a sophisticated cage of algorithmic surveillance. This paradox occurs when users praise the esthetic freedom of the AI while simultaneously labeling it a corporate robot. For example, a follower of Noonoouri commented: “I love your style, but are you a physical form or is this just an account with photoshop? I still don’t know what it is.” This comment expresses a proactive interpretation of the virtual style and lore (need satisfaction), while admitting the disruption of the narrative due to a lack of ontological clarity (need frustration). A follower of Lil Miquela commented on Miquela’s post posing with a real human model where followers compared them directly: “the robot more pretty.” This comment shows clear positive valuation and preference for the virtual persona (need satisfaction), while explicitly labeling Miquela a robot (need frustration). This presents a follower holding both admiration and acknowledgement of artificiality. The implications of these dynamics are multifaceted, presenting a stark contrast between psychological flourishing and distress. On the positive path, the integration of AI-generated imagery and anthropomorphized characteristics enhances user engagement by facilitating autonomy satisfaction. This is achieved through the aforementioned symbolic play, where users find opportunities for self-expression and creative exploration within the virtual narrative. By providing a stable yet interpretable framework, AI influencers lower the threshold for creative participation, effectively democratizing access to visual storytelling and promoting a sense of psychological ownership over digital content. In this state, users feel that their interaction is an authentic extension of their own agency, where the digital environment supports their intrinsic needs.

Conversely, a negative path emerges when the curtain is pulled back, and users encounter the reality of algorithmic orchestration. This exposure typically occurs during algorithmic failures, such as when a user receives highly repetitive, hyper-targeted ads or comments that feel invasive, or when the AI influencer’s responses become suspiciously formulaic. When a mismatch occurs between the user’s volitional engagement and the perception of being a target of data-driven manipulation, the sense of agency is rapidly eroded. This mismatch is typically not present at the onset of interaction; rather, followers often begin with a playful engagement with AI influencers. The disenchantment occurs when the cumulative weight of algorithmic nudges reaches a tipping point. At this juncture, the subconscious awareness of the AI’s artificiality shifts into a conscious realization of being targeted, triggering the algorithmic trap and shifting the user’s state from an active participant to a controlled object of a commercial script. Such experiences lead to profound autonomy frustration, manifesting as digital fatigue and psychological reactance. Rather than feeling empowered, users feel used by the system, which significantly undermines their digital well-being. This paradox underscores the urgent need for transparent and user-aligned design in generative AI systems, designs that meticulously balance algorithmic assistance with meaningful user control to ensure that creative agency is not merely a perceived illusion but a psychologically actualized reality (B. Chen et al., 2015; Ryan & Deci, 2017).

### 4.2. The Competence Paradox: From Scripted Mastery to Cognitive Dissonance

The data analysis reveals that the impact of AI influencers on user competence is initiated by the core features of AI influencers, specifically their highly customizable campaign structures and high domain knowledge. These attributes serve as the foundation for the user’s hedonic drive, characterized by the desire for trend-mastery, informed decision-making, and the pursuit of optimized lifestyle choices within the digital social sphere. This drive activates the central mechanism of smart consumerism, where the AI’s data-driven expertise and polished presentation ironically function as a structured pedagogical tool, where users treat the AI’s optimized content as a benchmark for personal proficiency. Because AI is perceived as an entity capable of processing vast amounts of information and presenting it with a level of precision unattainable by humans, users feel a sense of effectiveness in proactively adopting the AI’s curated insights to enhance their own digital presence and consumption habits. For example, Lu do Magalu often shares content that empowers users to make informed choices. Posts feature tutorials utilizing Magalu’s digital platforms or highlighting diverse product selections encourage users to explore and decide independently. Some examples of Magalu’s post include a post demonstrating how to navigate the Magalu app for personalized shopping experiences. Some follower comments include: “Loved the tips! Now I can find everything I need on my own [bre***],” and “Lu always helps me make better choices. Thank you! [lau***].”

According to SDT and the BPNSFS framework (B. Chen et al., 2015), regarding competence, interactions revealed users experiencing enhanced feelings of mastery when engaging with virtual influencers’ content. Comments like “Your tutorial helped me finally nail that look. I feel so confident now!” or “Thanks to your advice, it actually worked” demonstrate how aspirational posts enable users to feel effective. This process facilitates competence satisfaction; users perceive their interaction not merely as consumption, but as a proactive acquisition of mastery. The mechanism of smart consumerism represents a unique psychological process where users transform the scripted nature of AI, its pre-programmed and data-backed intelligence, into opportunities for self-enhancement. Within this framework, the AI’s authoritative voice and high domain knowledge do not function as overwhelming barriers; rather, they serve as a stable epistemic anchor that invites user learning and emulation. Because an AI influencer operates on logical optimization rather than human whim, it exists as a vessel of expertise. This perceived technical superiority allows users to engage in a form of vicarious mastery, where they proactively align their choices with AI’s perfect suggestions to feel more competent in their own social and esthetic lives. For example, Barbie is widely recognized for her expertise in fashion and lifestyle. When she participated in the #PillowChallenge, users praised her style and trend-setting ability, reinforcing the belief that she is treated as a social entity in the fashion domain. Similarly, Sophia the Robot is perceived as a credible voice in discussions on technology, AI ethics, and innovation, even though her responses are scripted. Sophia has utilized her own Instagram account as a technological showcase and has become more fashion-oriented. She has appeared in fashion shows and campaigns, suggesting she has influencer-like qualities. Sophia posted on her Instagram: “Making my Fashion Week debut! I’m so honored that designer Sadie Clayton chose a robot like me to model for her at Shanghai Fashion Week,” and “Love a good neckline, as long as it is breathable! As a learning robot, I’m always experimenting with fashion and trying out new looks. What do you think of this style?” Moreover, Lil Miquela or Shudu often share highly curated tutorial-style content or deep dives into fashion and technology that encourage followers to refine their own aesthetic skills. In posts where she explains the nuances of a new digital drop or a complex fashion trend, followers respond with a sense of increased self-efficacy. Comments often reflect this satisfaction: “I never understood how to style these pieces until I saw your breakdown [use***],” “Your tech reviews are so precise, I feel like a pro after watching [tec***],” “Following you makes me feel like I’m ahead of the trend curve [fas***],” and “The way you explain digital art makes me want to start my own project [art***].”

However, this satisfaction path is inherently countered by a parallel path of frustration when the same AI features are perceived as manipulative. The technical perfection that allows for scripted mastery can be sensed as a form of cognitive dissonance, where every interaction is recognized as an artificial construct designed to induce a false sense of proficiency for commercial gain. When users become aware that their perceived mastery is being steered by deceptive commercial scripts rather than genuine wisdom, the hedonic drive for growth is stifled, and the mechanism shifts toward cognitive dissonance. In this state, the sense of being an effective agent is replaced by the feeling of being an outperformed amateur, leading to competence frustration. The collapse into cognitive dissonance occurs at the intersection of perceived unattainability and algorithmic transparency. While traditional human influencers often trigger frustration through upward social comparison, where the problem is the user’s perceived biological or lifestyle deficit, the AI influencer introduces a unique ontological friction. The trigger is not merely the perfection of the image, but the realization that this perfection is a deceptive script optimized for commercial extraction. Unlike the human influencer, whose beauty is perceived as a trait, the virtual influencer’s perfection is decoded as an instrumental affordance designed to bypass consumer defenses. Consequently, the learning experience fails not because the user feels less than the influencer, but because they feel played by the system, paradoxically experiencing both potential empowerment and perceived predatory surveillance.

In posts featuring hyper-realistic but physically impossible standards, or overly promotional smart advice that serves a hidden sponsor, users express skepticism and self-doubt. Some of Noonoouri’s content may inadvertently lead to feelings of competence frustration among followers. For example, her posts often depict an idealized lifestyle, featuring flawless fashion and exclusive events, which may create a sense of unattainable standards for followers. Additionally, one of Bermuda’s followers expressed frustration that unrealistic, algorithmically perfect output by Bermuda sets unattainable standards: “I spent 10 years perfecting my guitar skills and putting bands together to make music, hustling my brains out to get more gigs at these little bars and am barely making my ends meet… and you’re literally created in a lab to churn out hit records and flaunt a lifestyle all over the internet… I mean like wow I halfway want to burn all of my guitars just quit. Ridiculous.” Another example, posts announcing a flash sale with minimal details about the products involve followers’ comments like: “It seems like it’s just advertising; I wanted more information about the products [gla***],” and “I miss more real interactions; everything seems very automated [oii***].” In addition, a caption like “Achieve this flawless look with my new AI-optimized skincare routine #ad” can trigger a realization of the gap between virtual and physical reality. In discussions from followers, comments reflect this frustration: “It’s easy to look like an expert when you’re literally made of pixels… it makes my real skin feel like a failure [sad***],” and “Is this real advice or just another algorithm trying to make me feel dumb so I buy more? [ske***].”

Thus, the AI influencer design presents a fundamental paradox of competence: it offers a platform for epistemic empowerment while simultaneously acting as a source of self-diminishment and cognitive alienation. This paradox reflects the tension between admiring the AI’s optimized appearance and feeling the dissonance of its technical repetition. For example, a follower of Lil Milquela commented: “Slay! … Why is your hair the same in every photo?” This comment shows appreciation for the leading trend and esthetic (need satisfaction), while expressing irritation at the AI-generated technical consistency that breaks the human illusion (need frustration). A follower of Lu do Magalu commented: “I love Lu, she’s so helpful! But sometimes the app feels like it’s just trying to force me to buy more than I need.” This comment presents that a follower utilized AI-driven insights for sophisticated consumption (need satisfaction), while acknowledging perceived manipulation of personal choice (need frustration). The implications of these dynamics present a stark contrast between intellectual flourishing and psychological inadequacy. On the positive path, the integration of high domain knowledge enhances user engagement by facilitating competence satisfaction through smart consumerism. By providing an optimized yet accessible framework of expertise, AI influencers lower the threshold for trend-mastery, democratizing access to sophisticated lifestyle insights and promoting a sense of psychological proficiency. Conversely, a negative path emerges when the script is exposed as a commercial deception. When a mismatch occurs between the user’s desire for growth and the perception of being a target of AI’s optimized perfection, the sense of efficacy is rapidly eroded. This realization triggers cognitive dissonance, leading to profound competence frustration, manifested as a sense of being outperformed by a machine. This paradox underscores the need for human-centric AI design that ensures digital expertise serves to elevate the user’s real-world competence rather than subordinating it to an artificial and unattainable standard (Vansteenkiste et al., 2020).

### 4.3. The Relatedness Paradox: From Simulated Intimacy to the Artificial Void

The data analysis indicates that the impact of AI influencers on user relatedness is rooted in the interplay between anthropomorphized characteristics and consistent social presence. These features act as a catalyst for the user’s hedonic drive for social connection, belonging, and the alleviation of loneliness within the digital ecosystem. This drive triggers the mechanism of parasocial enjoyment, where the AI’s strategic use of emotional storytelling and constant availability creates a persistent social presence that users treat as a legitimate foundation for a parasocial bond. Because the AI is accessible 24/7 and maintains a curated emotional narrative, users experience a sense of stable companionship, proactively integrating the virtual persona into their daily social routines.

According to SDT and the BPNSFS framework (B. Chen et al., 2015), for relatedness, users frequently expressed a sense of emotional closeness and companionship, treating virtual influencers as relational partners despite their synthetic nature. For example, comments such as “You always understand me better than my real friends” or “Talking to you feels like chatting with someone who truly gets me” (observed across high-engagement threads on personal storytelling posts) illustrate how consistent, non-judgmental responses from virtual influencers foster a perceived bond. These interactions often involved reciprocal-like exchanges, satisfying the need for belonging in a low-risk digital environment. This process fosters relatedness satisfaction; users perceive their interaction not as a mechanical exchange, but as a meaningful social attachment. The mechanism of parasocial enjoyment represents a specialized psychological process where users transform the simulated warmth of AI, its programmed responsiveness, and narrative consistency into opportunities for emotional connection. Within this framework, the AI’s lack of a physical body does not hinder closeness; instead, it serves as an emotional anchor that invites deep parasocial investment. Since the AI influencer is immune to human volatility and remains perpetually present, it exists as a reliable companion. This perceived availability allows users to engage in parasocial enjoyment, where they proactively bridge the gap between human and machine to satisfy their need for belonging. Lil Miquela frequently posts about personal challenges and emotions, prompting users to empathize and offer support. Lil Miquela shared: “I’m super grateful that my first experience with love was with someone who cared about me with his whole heart, and even through the embarrassing breakdown and breakup that followed, this legit feels like a breakthrough?” Rozy exhibits human-like expression and emotion, answers questions, and holds conversations, which could be leveraged in social media interactions with humans in a meaningful and engaging way. She posted her virtual ID card and her followers commented: “I can feel your frustration that you can’t vote by seeing your facial expression… [dai***],” and “Maybe Rozy is a real person existing somewhere in the world [sky***].” Rozy’s followers treated Rozy like a real human: “Where is this place? Aren’t you hot? Your skin will be burnt by the sun [gro***].” Many users post messages of support and their loyalty to their AI influencers, such as “We love you, Miquela!” or “Sophia, you are so inspiring!”, illustrating the depth of their emotional connection.

Drawing on the hedonic drive for connection, parasocial enjoyment occurs when users treat the AI’s ongoing life story as a shared journey. In this process, the user becomes an active participant in a digital friendship, internalizing the AI’s experiences as part of their own social world. This proactive engagement constitutes a high level of psychological relatedness, as the user feels recognized and accompanied by a consistent presence. Consequently, this leads to relatedness satisfaction, fulfilling the basic psychological need to feel connected to others (Ryan & Deci, 2017). By navigating the boundaries of this virtual bond, users experience a state of simulated intimacy, feeling socially embedded even in the absence of a human peer. Lil Miquela’s profile, for instance, frequently features intimate diary-like captions and interactions that simulate a vulnerable, human-like social life. In posts where she shares her struggles or friendships, followers often respond with deep empathy and communal support. Comments often highlight this bond: “Seeing your posts makes me feel like I’m not alone in feeling this way [lon**],” “You’re more real than half the people I know in real life [fri***],” “I feel like we’ve grown up together over the years [loy***],” and “Thanks for always being here when I need a distraction [com***].” “Whether you are a human or not, we still love you. We are your fans. Never give up. We love you Miquela” [Follower of Lil Miquela], “I am very proud of you, I love you so much” [Follower of Lil Miquela], “I love you my goddess angel” [Follower of Noonoouri], “I’m feeling this vibe!” “So inspiring!” [Follower of Noonoouri], “Love you, dear my friend” [Follower of Rozy], “You have Harley Davidson. I really appreciate you. You are super happy women,” [Follower of Rozy] and “Will you be my girlfriend in magical world…?” [Follower of Rozy].

However, this satisfaction path is inherently countered by a parallel path of frustration when the same AI features are viewed as a manifestation of underlying isolation. The emotional storytelling that builds intimacy can be perceived as an artificial void, a realization that the warmth is an automated output devoid of genuine reciprocity or shared consciousness. When users encounter the limits of AI’s empathy, such as its inability to provide personalized, non-scripted emotional support, the hedonic drive for connection is stifled, and the mechanism shifts toward social alienation. In this state, the sense of being accompanied is replaced by the hollow feeling of interacting with a script, leading to relatedness frustration. While relatedness frustration can also occur in human influencer-follower dyads, often triggered by perceived neglect or commercial insincerity, the frustration elicited by AI influencers is ontologically distinct. In human interactions, the follower’s disappointment stems from a refusal to reciprocate available empathy. Conversely, in AI-driven interactions, the frustration arises from the absence of the capacity to empathize. When users encounter the limits of an AI’s synthetic empathy, manifesting as repetitive, non-scripted emotional voids, the hedonic drive for connection is not merely ignored but stifled by the realization of the script. This creates a paradoxical experience where the user’s pursuit of social accompaniment exists in a state of constant friction with the reality of mechanical interaction, ultimately transforming the desire for intimacy into a profound state of social alienation. The transition from satisfaction to frustration often occurs at reciprocity failure, the moment when a user’s genuine emotional vulnerability is met with a generic, commercially motivated bot response or a promotional pivot. In moments where AI’s feelings are exposed as marketing tools, or when it fails to respond authentically to serious user interactions, followers express profound disillusionment. For example, a vulnerable post about mental health followed by an automated #ad or a generic “Love you all!” comment can trigger a sharp realization of AI’s ontological emptiness. In follower discussions, comments reflect this frustration: “I poured my heart out in the DMs and got a discount code… it’s a cold reminder that you’re just a bot [emp***],” and “It’s scary how much I cared about a character that literally can’t feel anything back. I feel more alone now [voi***].”

Thus, the AI influencer design presents a fundamental paradox of relatedness: it offers a platform for simulated companionship while simultaneously exposing the user to a deeper sense of technological solitude. This paradox is characterized by a user’s emotional investment in the AI’s vulnerability, immediately followed or paired with a realization of its mechanical nature. A follower of Lil Miquela commented: “So you dated a real person? … You are a robot.” This comment shows investment in the human-like drama of Miquela’s relationship (need satisfaction), while confronting her digital unreality in the same breath (need frustration). Another follower commented: “I know this is crazy but I believe you.” This comment presents emotional belief and attachment to the narrative (need satisfaction), while expressing self-aware dissonance (“this is crazy”), acknowledging the absurdity of the bond (need frustration). The implications of these dynamics reveal a contrast between digital belonging and emotional vacancy. On the positive path, the use of anthropomorphized storytelling facilitates relatedness satisfaction through parasocial enjoyment. By providing a stable and emotionally resonant presence, AI influencers lower the barrier to social engagement, promoting a sense of psychological inclusion in a virtual community. Conversely, a negative path emerges when the simulated bond is revealed as a mechanical facade. When a mismatch occurs between the user’s need for empathy and the reality of the artificial void, the sense of connection is rapidly eroded. This realization triggers relatedness frustration, manifested as a heightened sense of isolation. These boundaries are often disrespected through scripted vulnerability, where AI influencers, such as Lil Miquela and Rozy, simulate deeply personal human experiences like identity crises, social activism, or existential grief to drive commercial engagement. For instance, Rozy’s participation in zero-waste campaigns functions as a brand-safe performance of activism; while she mimics the outward signs of environmental concern, her inability to experience the physical consequences of the crisis she promotes reveals an ontological void.

## 5. Discussion and Implications

### 5.1. Theoretical Implications

This study is one of the earliest studies to explore the rising phenomenon of AI influencers. Its primary theoretical contributions can be categorized into three key areas. First, the study enriches literature by developing an empirically grounded theoretical framework that illustrates how the three psychological mechanisms underlie the influence of AI influencers on users’ psychological needs. This research builds on the work of Wan et al.’s (2024) work, which identified that virtual influencers’ effectiveness in promoting certain behaviors depends on their level of anthropomorphism. This study contrasts from previous studies on human influencers in some distinct areas. In human influencer studies, self-expression is often discussed in the context of identity alignment, with consumers following influencers who reflect their personal values, lifestyles, and aspirations. AI influencers, despite lacking personal experiences, enable self-expression in novel ways. Their digitally generated nature allows users to project their own ideals onto them, treating AI influencers as blank canvases rather than fully formed personalities. Moreover, AI influencers often operate within customizable virtual environments, allowing for a greater degree of audience co-creation compared to human influencers. This fluidity may explain why some users feel more comfortable engaging with AI influencers as a means of expressing their digital identity, free from the constraints of real-world social norms. While AI-generated imagery supports autonomy by enabling identity experimentation and self-projection, it may simultaneously restrict it by reinforcing the illusion of control within pre-engineered digital boundaries. This research builds on the work of Gutuleac et al. (2024), who identified that highly anthropomorphized virtual influencers can trigger consumer discomfort due to uncanniness, but the presence of social cues can reduce this effect, highlighting dual implications of the same feature.

Moreover, source credibility, including expertise, trustworthiness, and attractiveness, has been a critical determinant of influencer effectiveness in prior studies (Reinikainen et al., 2020). Human influencers derive credibility from their expertise, real-life experiences, and social proof, such as peer validation and past achievements. Conversely, AI influencers establish credibility through a different mechanism. The present study indicates that their perceived expertise is often constructed through algorithmic, data-driven optimization and their association with authoritative brands. Additionally, the non-human nature of AI influencers means they are less prone to scandals, impulsive behaviors, or biases, which can enhance perceptions of their objectivity. However, this credibility is conditional; users may still question the legitimacy of AI influencers, as their endorsements are pre-programmed rather than personally motivated. While human influencers also face skepticism regarding their commercial sincerity, often dismissed as being only in it for the money, the doubt directed toward AI is ontologically distinct. For example, when a human influencer promotes a skincare product, the audience evaluates whether they actually use it; for an AI influencer like Rozy, the audience recognizes that use is a physical impossibility. This shift from questioning a person’s motives to questioning an entity’s very capacity to endorse creates a unique credibility gap that cannot be bridged by mere visual realism (Liu et al., 2025).

Additionally, parasocial relationships, or one-sided emotional bonds with influencers, have been studied in the context of human influencers. Prior research suggests that these relationships are strengthened through perceived authenticity, relatability, and personal engagement (e.g., Ko & Wu, 2017; Munnukka et al., 2019). However, AI influencers, by their nature, lack genuine emotions and lived experiences, yet users still form strong parasocial relationships with them. The present study suggests that the perceived consistency and availability of AI influencers may compensate for the lack of human authenticity (Allal-Chérif et al., 2024). Unlike human influencers, who may experience controversies, inconsistencies, or personal struggles that weaken audience trust, AI influencers provide a stable and controlled engagement experience. This stability appears to foster a different form of parasocial bond—one rooted in reliability and idealized perfection rather than relatability.

Second, this study extends the application of SDT into the realm of human–AI interaction by revealing how AI influencers can simultaneously satisfy and frustrate users’ basic psychological needs and what the potential implications are. The findings reveal the dual nature of AI influencers: they can meaningfully fulfill users’ psychological needs for autonomy, competence, and relatedness, yet also risk undermining these needs when their design becomes overly controlled, commercialized, or emotionally inauthentic. While prior literature has primarily focused on human influencers, traditional media, and how human interactions and social environments support or thwart need satisfaction (Deci & Ryan, 2000), this research highlights the unique ways in which AI-driven personas evoke complex psychological responses and implications. Notably, the duality of need satisfaction and frustration caused by AI influencers challenges the assumption that psychological needs are either fully supported or thwarted. This research builds on Marriott and Pitardi’s (2024) study that highlights a paradox of AI friendship by identifying both the positive and negative implications of AI influencer–user engagement. This study supports a more dynamic understanding, where the same AI influencer feature can empower users by enabling autonomy, while simultaneously frustrating them by limiting perceived authenticity or control. This duality highlights the importance of responsible AI influencer design that balances engagement with user well-being.

In addition, the emergence of dual psychological outcomes—satisfaction and frustration—is rooted in the inherent tension between AI’s anthropomorphized agency and its algorithmic essence. Grounded in SDT (Ryan & Deci, 2017), this study posits that users engage with AI influencers not merely as marketing tools, but as AI-mediated social agents that enter their motivational ecosystems. The dual-path process occurs because the technological affordances of AI are fundamentally paradoxical. They provide the consistent presence required for need satisfaction while simultaneously embodying the structural asymmetries that lead to need frustration. According to paradox theory (Smith, 2014), these outcomes are opposing yet connected components that coexist within the human–AI interaction. The satisfaction path (e.g., symbolic play) thrives when users accept the pact of fiction, allowing them to proactively project their own values onto the AI’s stable semiotic anchor. However, the frustration path (e.g., algorithmic trap) is triggered when the linkage between the user and the agent is exposed as a commercial orchestration. In this moment, the clashing dynamics of human-like appearance versus machine-like control produce an inherent strain that shifts the user’s experience from psychological flourishing to digital alienation.

### 5.2. Practical Implications

This study offers several practical implications for diverse stakeholder groups. First, the findings of this study highlight the need for AI influencer designers and social media strategists to develop their design strategies that support users’ psychological needs. For AI influencers to be effective, their design and social media engagement strategies should be optimized to fulfil users’ psychological needs while maintaining trust and relatability. Developers can enhance autonomy by enabling users to personalize their interactions with AI influencers, such as selecting engagement frequency, conversational styles, or content preferences. To foster competence, AI influencers can be designed to provide skill-enhancing, informative, and motivational content that empowers users and supports them in attaining personal and consumer goals. Clearly disclosing AI-generated content and ensuring ethical communication can help build credibility and foster user trust. Relatedness can be reinforced by incorporating emotional intelligence, allowing AI influencers to engage in more meaningful and socially satisfying interactions. For example, brands can incorporate storytelling techniques that make AI influencers more compelling and emotionally resonant. By integrating these designs and strategies, AI influencers can become more effective in meeting users’ psychological needs while retaining strong and lasting connections with their audience and followers. However, this surgical level of audience targeting carries the inherent risk of a psychological backfire. Because AI influencers are designed to mimic natural, intuitive, and emotionally engaging human interactions, they establish high expectations for social reciprocity (Arsenyan & Mirowska, 2021). When the AI inevitably reaches its empathic limit, the point where it cannot provide personalized, non-scripted emotional support, the user’s initially satisfied drive for connection is stifled (Sands et al., 2022b). This reciprocity failure can transform the perceived social presence into a hollow, mechanical interaction, leading to relatedness frustration and a heightened sense of digital alienation (Ryan & Deci, 2017). Thus, designers must balance personalized engagement with ontological disclosure to ensure that virtual presence complements rather than deceptively substitutes for authentic human reciprocity.

Second, this study presents opportunities for brands and businesses to enhance customer engagement and personalization. To maximize user engagement and psychological fulfillment, brands can leverage user data and AI-driven customization. For instance, AI influencers can be designed to deliver personalized interactions tailored to individual preferences, behavioral patterns, and psychological needs. By leveraging user data, AI influencers can adjust their content, engagement style, and tone to align with each user’s interests and communication preferences. This level of personalization enhances user satisfaction by making interactions feel more meaningful and relevant, fostering a deeper sense of connection. Additionally, when AI influencers offer personally relevant content that aligns with users’ motivations, users are more likely to engage with the brand’s messaging. By making AI influencers more adaptive and responsive, brands can strengthen audience trust and encourage long-term engagement.

Third, these paradoxical experiences emphasize the necessity for ethically grounded AI narratives that respect the boundaries of human emotion, ensuring that virtual presence serves to complement, rather than substitute or devalue, the profound need for authentic human reciprocity (B. Chen et al., 2015; Tukachinsky, 2010). Achieving this in practical terms does not require controlling the full range of uncontrollable human emotional triggers or interpretations, an inherently impossible task given the diversity of individual responses. Instead, developers can implement ontological disclosure (De Cremer & Kasparov, 2022), such as persistent, context-aware signals (e.g., disclaimers in bios/profiles, periodic textual/watermark cues like “I am a virtual/synthetic entity,” or visual indicators during emotionally intense interactions) that explicitly remind users of the AI’s non-human, non-reciprocal nature and thereby reduce deceptive anthropomorphism without eliminating emotional engagement. Complementing this, empathic guardrails can be programmed as rule-based or AI-monitored triggers, for instance, when sentiment analysis or keyword patterns detect elevated emotional vulnerability (e.g., expressions of loneliness, distress, or dependency), the system can gently redirect the user toward human support resources (e.g., “This conversation is meaningful, but for deeper support, consider reaching out to a friend or professional helpline. Here’s how…”), introduce intentional friction (e.g., response delays, reflective prompts about real-world connections, or limits on prolonged affective mirroring), or cap certain response types. These mechanisms, relevant to responsible AI design for companions and chatbots, help virtual entities provide a supportive presence while safeguarding the unique sanctity of genuine human connection. Implementation requires interdisciplinary collaboration (designers, ethicists, psychologists) and ongoing testing for effectiveness and unintended effects.

## 6. Conclusions

This study examined how AI influencers, through their distinct features and psychological mechanisms, shape users’ psychological experiences of autonomy, competence, and relatedness. By analyzing three key mechanisms, this research revealed the dual impacts of AI influencer features on user motivation. While the symbolic identity of AI personas enables self-expression, it can also impose limits on interpretive freedom. Similarly, the strategic storytelling in brand partnerships enhances users’ sense of mastery, yet may hinder independent judgment. Finally, anthropomorphized characteristics foster a powerful illusion of social connection, but risk emotional disillusionment over time. These findings underscore the need for critical design approaches that do not merely optimize engagement but also support users’ psychological well-being. As AI influencers become increasingly integrated into digital culture and commerce, designers and marketers should consider both the empowering and potentially undermining effects these entities have on users’ fundamental psychological needs.

Several avenues for future research emerge. To strengthen the interpretation of these findings, alternative factors should be considered in future studies such as baseline trust in AI, prior exposure to automation, and familiarity with digital influencer marketing (Alboqami, 2023; De Cicco et al., 2024). Users with higher AI readiness or positive dispositional attitudes toward automation may be more predisposed to perceive the AI’s data-driven precision as Competence Satisfaction rather than an Algorithmic Trap, while those with lower trust may experience heightened Autonomy Frustration regardless of the narrative quality. Furthermore, the novelty effect of virtual entities tends to diminish with frequent exposure; AI-savvy digital natives often apply a more utilitarian lens, effectively navigating the pact of fiction to avoid Relatedness Frustration by grounding their expectations in functional rather than emotional reciprocity (Arsenyan & Mirowska, 2021). Finally, a user’s past experiences with the perceived insincerity of human influencers can moderate their judgment, leading them to value the honest fakery and ontological stability of AI as a more reliable alternative, suggesting that paradoxical reactions are often a relative evaluation of source credibility (Allal-Chérif et al., 2024; Block & Lovegrove, 2021). Future studies could also explore the long-term psychological impact of interacting with AI influencers, particularly whether prolonged exposure to AI influencers affects self-esteem, social behaviors, or the way users engage with human influencers. Moreover, while AI influencers are growing in popularity, their effectiveness compared to human influencers remains underexplored. Future studies could conduct comparative studies to analyze differences in emotional engagement, trust formation, and persuasion effectiveness between AI and human influencers. Lastly, since cultural values shape how people perceive technology, AI influencers may be received differently across cultures. Future research could investigate how cultural differences influence users’ engagement with AI influencers, providing insights for brands targeting global markets.

## Figures and Tables

**Table 1:** Overview of Selected Previous Studies on AI Influencers.

Reference	Key Findings	Method	Research Gaps and Contributions of This Study
Alboqami (2023)	Explores the recipes that can drive high customer trust in AI influencers. A configuration of source attractiveness, source credibility, and congruencies acts as a driver of consumers’ trust in an AI influencer.	Fuzzy set qualitative comparative analysis	Research gaps: Predominantly focused on users’ acceptance, trust, and attitudinal responses, treating AI influencers primarily as communication or marketing instruments rather than as social entities that shape psychological experience. Fragmented integrative insight into how users psychologically experience interactions with AI influencers. A lack of understanding of how AI influencers satisfy and frustrate users’ basic psychological needs, and how these paradoxical dynamics influence users’ psychological well-being. Contributions of this study: Advances understanding of AI influencer–user interaction by examining how AI influencers function as AI-mediated social agents that shape users’ intrinsic motivation. Identifies key psychological mechanisms through which AI influencer design and communication features influence users’ experiences of autonomy, competence, and relatedness. Extends SDT to the context of AI-mediated social interaction, highlighting the complex implications of AI influencers for users’ psychological well-being.
Arsenyan and Mirowska (2021)	Examines virtual agents’ similarity to humans in terms of behavior and how reactions differ among types of influencers. Human-like virtual influencer receives significantly lower positive reactions from consumers.	Data scraping	
De Cicco et al. (2024)	Explores users’ perceptions of virtual influencers participating in sponsorship activities on Instagram. Examines how the influencer’s nature (human vs. virtual) impacts perceptions and investigates potential interaction effects of the type of message conveyed (hedonic vs. utilitarian) on the influencer and brand-related variables.	Experimental study	
Feng et al. (2024)	Develops an AI influencer attributes scale, consisting of key measures of AI influencers’ perceived attributes, and unveils the relationship between each attribute and consumers’ acceptance of AI influencers as product/brand endorsers. Identifies seven key attributes of AI influencers, which are anthropomorphism, artificiality, attractiveness, luminary, quality, trendiness, and robophobia.	Mixed method	
Block and Lovegrove (2021)	Analyzes an AI influencer’s communication to identify what makes it appealing to audience. The influencer’s identity and messaging effectively integrate into a single strategic communication tool, whereby the influencer is the strategy and the message.	Textual and sentiment analysis	
J. Park et al. (2024)	Investigates how people perceive and evaluate AI-generated content and accounts compared to human-created accounts and content. Identifies that participants perceived the AI and influencer accounts as more attractive than the public account.	Survey and interviews	
Sands et al. (2022a)	Examines how consumers respond to social media influencers that are created through AI and compares effects to human influencers. Compares AI and human influencers to explore consumer perceptions, and finds similarities (following intentions, perceived personalization) and differences (AI lowers source trust and increases WOM).	Experiment study	
Thomas and Fowler (2021)	Examines the implications of using AI influencers and sets out to provide guidance for when controversies arise from AI influencers. AI influencers can produce positive brand benefits like those produced by human celebrity endorsers.	Experiment study	

**Table 2:** The Paradox of Psychological Needs.

Dimension	Hedonic Drive	Need Satisfaction	Need Frustration
Autonomy	Volitional Enjoyment	Symbolic Play: Proactive interpretation of virtual narratives and lore.	Algorithmic Trap: Perceived manipulation of personal taste and choices.
Competence	Leading Trends	Smart Consumerism: Sophisticated consumption utilizing AI-driven insights.	Cognitive Dissonance: Sense of inferiority compared to the AI’s optimized perfection.
Relatedness	Alleviating Loneliness	Parasocial Enjoyment: Sense of belonging through simulated intimacy.	Artificial Void: Disillusionment from inauthentic, mechanical responses.

## Data Availability

Data available in a publicly accessible repository. The original data presented in the study are openly available in FigShare at https://figshare.com/s/ea115292ff56c322e935 accessed on 29 January 2026.
